# MDD-Palm: Identification of protein *S*-palmitoylation sites with substrate motifs based on maximal dependence decomposition

**DOI:** 10.1371/journal.pone.0179529

**Published:** 2017-06-29

**Authors:** Shun-Long Weng, Hui-Ju Kao, Chien-Hsun Huang, Tzong-Yi Lee

**Affiliations:** 1 Department of Medicine, Mackay Medical College, New Taipei City, Taiwan; 2 Department of Obstetrics and Gynecology, Hsinchu Mackay Memorial Hospital, Hsinchu city, Taiwan; 3 Mackay Junior College of Medicine, Nursing and Management, Taipei, Taiwan; 4 Department of Computer Science and Engineering, Yuan Ze University, Taoyuan, Taiwan; 5 Tao-Yuan Hospital, Ministry of Health & Welfare, Taoyuan, Taiwan; 6 Innovation Center for Big Data and Digital Convergence, Yuan Ze University, Taoyuan, Taiwan; Huazhong University of Science and Technology, CHINA

## Abstract

*S*-palmitoylation, the covalent attachment of 16-carbon palmitic acids to a cysteine residue via a thioester linkage, is an important reversible lipid modification that plays a regulatory role in a variety of physiological and biological processes. As the number of experimentally identified *S*-palmitoylated peptides increases, it is imperative to investigate substrate motifs to facilitate the study of protein *S*-palmitoylation. Based on 710 non-homologous *S*-palmitoylation sites obtained from published databases and the literature, we carried out a bioinformatics investigation of *S*-palmitoylation sites based on amino acid composition. Two Sample Logo indicates that positively charged and polar amino acids surrounding *S*-palmitoylated sites may be associated with the substrate site specificity of protein *S*-palmitoylation. Additionally, maximal dependence decomposition (MDD) was applied to explore the motif signatures of *S*-palmitoylation sites by categorizing a large-scale dataset into subgroups with statistically significant conservation of amino acids. Single features such as amino acid composition (AAC), amino acid pair composition (AAPC), position specific scoring matrix (PSSM), position weight matrix (PWM), amino acid substitution matrix (BLOSUM62), and accessible surface area (ASA) were considered, along with the effectiveness of incorporating MDD-identified substrate motifs into a two-layered prediction model. Evaluation by five-fold cross-validation showed that a hybrid of AAC and PSSM performs best at discriminating between *S*-palmitoylation and non-*S*-palmitoylation sites, according to the support vector machine (SVM). The two-layered SVM model integrating MDD-identified substrate motifs performed well, with a sensitivity of 0.79, specificity of 0.80, accuracy of 0.80, and Matthews Correlation Coefficient (MCC) value of 0.45. Using an independent testing dataset (613 *S*-palmitoylated and 5412 non-*S*-palmitoylated sites) obtained from the literature, we demonstrated that the two-layered SVM model could outperform other prediction tools, yielding a balanced sensitivity and specificity of 0.690 and 0.694, respectively. This two-layered SVM model has been implemented as a web-based system (MDD-Palm), which is now freely available at http://csb.cse.yzu.edu.tw/MDDPalm/.

## Introduction

*S*-palmitoylation (also known as *S*-acylation) is an important reversible lipid modification of proteins that involves the covalent attachment of 16-carbon palmitic acid to a cysteine residue via thioester linkage [1–5]. *S*-palmitoylation plays a significant role in regulating protein trafficking and protein-protein interaction by modifying the target cysteine residues on proteins [6–8]. Protein *S*-palmitoylation is also associated with a variety of physiological and biological processes including neuronal development, signal transduction, apoptosis, mitosis, etc. [6–9]. Additionally, protein *S*-palmitoylation plays a regulatory role in various diseases including Huntington’s disease [10], type 1 diabetes with ZDHHC17 [11], and amyloidosis, alopecia, and osteoporosis with ZDHHC13 [12]. Although mass spectrometry (MS)-based proteomics can be used to identify an increasing number of *S*-palmitoylated peptides [1,3,13,14], the experimental identification of large-scale *S*-palmitoylated proteomes is still time-consuming and labor-intensive. Moreover, owing to the labile nature and low abundance of *in vivo S*-palmitoylation sites, their detailed characteristics and mechanisms need to be clarified. However, previous studies have reported that there are no consensus motifs for *S*-palmitoylation substrate sites [2–4,15]. Therefore, designing an effective method to explore the potential substrate motifs of protein *S*-palmitoylation sites is an urgent demand in bioinformatics.

Owing to the biological importance of protein *S*-palmitoylation, several tools for predicting protein *S*-palmitoylation sites have been created in recent years. CSS-Palm, which was developed by Zhou et al. [16,17], is the first *S*-palmitoylation site predictor to use a clustering and scoring strategy based on the BLOSUM62 matrix. The prediction performance of CSS-Palm is encouraging, with highly positive jackknife validation results (sensitivity 82.16% and specificity 83.17% for cut-off score 2.6). CSS-Palm 4.0 reportedly offers a significant improvement in performance as compared to the previous version. NBA-Palm, which was designed by Xue et al. [18], uses the Naive Bayes algorithm to predict palmitoylation sites. Another predictor, CKSAAP-Palm, was created by Wang et al. [19] based on the Composition of K-Spaced Amino Acid Pairs (CKSAAP). In addition, Hu et al. [20] developed IFS-Palm, which achieved significant improvement in predicting *S*-palmitoylation sites. Shi et al. [21] proposed the WAP-Palm prediction tool based on a multiple feature extraction method. Recently, a new prediction tool, PalmPred, was reported by Kumari et al. [22] to yield an accuracy of 91.98% and a Matthews Correlation Coefficient (MCC) of 0.71%.

The present study focuses on the identification of *S*-palmitoylation sites with potential substrate motifs. Based on the *in silico* characterization of substrate sites, sequence-based features including amino acid composition (AAC), amino acid pair composition (AAPC), position specific scoring matrix (PSSM), position weight matrix (PWM), amino acid substitution matrix (BLOSUM62), and accessible surface area (ASA) were selected to discriminate between *S*-palmitoylation sites and non-*S*-palmitoylation sites. Additionally, we applied maximal dependence decomposition (MDD) [23] to explore promising consensus motifs for *S*-palmitoylation sites. MDD can moderate large-scale *S*-palmitoylation data into subgroups according to the maximal dependencies of amino acid composition surrounding the substrate sites of *S*-palmitoylation. Consequently, a support vector machine (SVM) was utilized to build a predictive model for each subgroup containing MDD-identified substrate motifs. Furthermore, an independent testing dataset, which was truly blind to the training dataset, was extracted from the experimental data of published literature in order to demonstrate the effectiveness of the proposed models in the evaluation of five-fold cross-validation. To facilitate the study of protein *S*-palmitoylation, MDD-identified substrate motifs were employed to implement a web-based tool called MDD-Palm (http://csb.cse.yzu.edu.tw/MDDPalm/) for identifying *S*-palmitoylation sites and their corresponding motifs.

## Material and methods

### Construction of training and testing datasets

The experimentally verified *S*-palmitoylation sites were collected from dbPTM 3.0 [24,25], which contains 498, 107, 43, and 109 *S*-palmitoylation sites in human, mouse, rat and other species, respectively. In addition, based on a literature survey of the PubMed database, a total of 856 *S*-palmitoylation sites were manually extracted from two research articles: Yang et al. [26] identified 427 human proteins with 790 *S*-palmitoylation sites and Forrester et al. [27] identified 66 *S*-palmitoylation sites on 51 human proteins. This study was intended at exploring potential substrate motifs based on the amino acids surrounding the *S*-palmitoylated cysteines. Thus, a window length of 2*n* + 1 was utilized to extract sequence fragments centered at the experimentally verified *S*-palmitoylation sites; it contained *n* upstream and *n* downstream flanking amino acids. Sequence fragments with a window length of 2*n* + 1 amino acids centered at the *S*-palmitoylated cysteine residue were regarded as the positive training dataset. Sequence fragments centered at the cysteine residue without the annotation of *S*-palmitoylation were regarded as the negative training dataset. Based on a window size of 21 (*n* = 10), the training dataset comprised 1294 positive data and 11,646 negative data.

In an attempt to avoid overestimating predictive performance, the CD-HIT program [28] was employed to remove homologous sequence fragments from the positive and negative datasets. CD-HIT is an effective tool for clustering protein sequences based on a specified sequence similarity value. One sequence was chosen to represent each cluster. Owing to the incomplete information about experimentally validated *S*-palmitoylation sites, based on the analysis of sequence fragments, a negative sequence might sometimes appear identical to a positive sequence, potentially causing false positive or false negative predictions. Therefore, CD-HIT was applied again by running cd-hit-2d across the positive and negative training data with 100% sequence identity [29]. If a sequence in the negative set was the same as a sequence in the positive set, only the sequence in the positive set was reserved, and the sequence in the negative set was discarded. After filtering out homologous fragments with 50% sequence identity (by running cd-hit and psi-cd-hit) as shown in Table 1, the combined non-homologous training dataset comprised 710 positive sequences and 5676 negative sequences.

**Table 1:** Data resource and statistics of training and independent testing dataset.

Dataset	Resource	Species	Number of *S*-palmitoylation sites (Positive data)	Number of *non*-*S*-palmitoylation sites (Negative data)
**Training data**	dbPTM 3.0	Human	498	4,671
		Mouse	107	1,822
		Rat	43	603
		Others	109	1,279
	Forrester MT *et al*. (PMID: 21044946)	Human	66	1,036
	Yang W *et al*. (PMID: 19801377)	Human	790	8,533
	**Combined non-homologous dataset**	All	710	5,676
**Independent testing data**	Gould *et al*. (PMID: 26165157)	Mouse	613	5,412

Based on the binary classification of *S*-palmitoylation and non-*S*-palmitoylation sites, the positive and negative datasets were used to build up a predictive model. Five-fold cross-validation was then applied to evaluate how well it distinguished between positive and negative datasets. With the optimization of parameters in the predictive model, however, the predictive performance might be overestimated because of over-fitting of the training dataset [29]. To assess the actual predictive performance of the proposed models, a blind independent test set was generated. The dataset for independent testing was generated by manually extracting *S*-palmitoylated peptides from Gould’s research [30] based on site-specific proteomic mapping of cysteine modification. Similar to the training dataset, based on a window size of 21 (*n* = 10), the independent testing dataset contained 613 positive and 5412 negative sequences (Table 1).

### Investigation and encoding of training features

This study emphasized the investigation of sequence-based features such as amino acid composition (AAC), amino acid pair composition (AAPC), position specific scoring matrix (PSSM), position weight matrix (PWM), amino acid substitution matrix (BLOSUM62), and accessible surface area (ASA). To create an SVM prediction model, fragment sequences must be transformed into numeric vectors according to various features. Orthogonal binary coding is one of the most popular methods of converting amino acids into numeric vectors known as 20D binary code [31]. The number of feature vectors was (2*n* + 1) × 20 to represent the flanking amino acids surrounding the *S*-palmitoylation sites. The training dataset contains *k* vectors {*x*_*i*_, *i* = 1, 2 …, *k*} which are corresponding to the *k* sequence fragments along with a specified window length. To classify the positive and negative data, a label was applied to each vector to mark the class of its corresponding protein. For composition of amino acids around the *S*-palmitoylation sites, the vector *x*_*i*_ had 21 elements for AAC and 441 elements for AAPC. Some rare amino acids and non-existing “X” residues were used to represent less than 21-mer fragment sequences at an N- or C-terminus [32].

The amino acid substitution matrix (BLOSUM62) was built on the alignments of amino acid sequences with no more than 62% identity between two peptide sequences. Fragmented sequences with a window length of 21 amino acids can be encoded as numeric vectors based on the substitution scores of 20 amino acids in BLOSUM62. With reference to the SulfoSite method [33], the PWM was determined using non-homologous training data. The PWM describes the frequency of occurrence of amino acids surrounding the *S*-palmitoylation sites, and was utilized in encoding the fragment sequences. Each residue of a training dataset was represented by a matrix of *m × w* elements, where *w* is a window size equal to 21, and *m* represents 21 elements including 20 types of amino acids and one non-existent signal “X”.

Position Specific Scoring Matrix (PSSM) is an effective sequence feature that has been widely used for the prediction of subcellular localization, protein secondary structures, and protein function sites [34–37]. In this work, PSSM profiles were generated by a PSI-BLAST [38] search against a non-redundant database of *S*-palmitoylated sequences. The score values of the PSSM profile represent the multiple sequence alignments of proteins that may have structures similar to different amino acid compositions. Extracting from the PSSM profile, the matrix of (2*n* + 1) × 20 elements had rows centered on the substrate site, where 2*n* + 1 represents the window size and 20 is the number of position-specific scores for each type of amino acid. Next, the (2*n* + 1) × 20 matrix was transformed into a 20 × 20 matrix by summing up the rows that were associated with the same amino acid type. Then, each element in 20 × 20 matrix was divided by window length 2*n* + 1 and normalized using the following formula: $\frac{1}{1+e^{-x}}$ [39].

The structural features of ASA were investigated based on the accessibility of a side-chain of amino acids on the surface of a protein that experienced post-translational modification [40]. RVP-Net [41] was used to calculate the ASA value from the protein sequence because of the lack of most *S*-palmitoylated protein tertiary structures in PDB [42]. RVP-Net can predict the real ASA of a residue based on information about the neighborhood using a neural network. The value of ASA was the percentage of solvent-accessible area of each amino acid on the protein. The full-length protein sequences were used as input data for RVP-Net to compute the ASA value of all of the residues. The ASA values of amino acids around the *S*-palmitoylation sites were then extracted and normalized to the range 0–1. In addition to the investigation of single features, hybrid features were formed by combining the best feature with other single features. Based on the cross-validation performance of each feature, the single feature with the best performance was incorporated along with other single features to enhance predictive power. S1 Fig describes the conceptual flowchart for combining the PSSM and BLOSUM62 features for each sequence fragment. Before construction of the SVM classifier, all numeric data needed to be scaled into values ranging from −1 to +1 to improve prediction effectiveness.

### Detection of substrate motifs by maximal dependence decomposition

Previous studies [1,15] have reported that *S*-palmitoylation can be catalyzed by palmitoyltransferases (PATs), which are composed of 23 PAT enzymes defined by the presence of an aspartate-histidine-histidine-cysteine (DHHC) motif. Although DHHC enzymes display substrate specificity, a substrate can be palmitoylated by one or more enzymes; e.g., huntingtin can be palmitoylated by DHHC17 and DHHC13 [43] and SNAP-25 by DHHC2, DHHC3, DHHC5, DHHC15, and DHHC17 [10]. As the number of experimentally identified *S*-palmitoylation peptides has increased, the investigation of substrate motifs to facilitate the study of protein *S*-palmitoylation is becoming imperative. Although a number of tools for predicting *S*-palmitoylation sites have been developed, their ability to identify *S*-palmitoylated sites and their corresponding substrate motifs is limited. Thus, the aim of this study was to explore motif signatures of protein *S*-palmitoylation based on the amino acids surrounding substrate sites. Maximal dependence decomposition (MDD) [44] was utilized to cluster all fragment sequences into subgroups in order to detect the statistically conserved motifs among large-scale sequence data. The clustering method was performed using MDDLogo [23], which demonstrated the effectiveness of dividing a group of protein sequences into smaller subgroups before the computational identification of PTM sites [31,45–55].

As presented in S2 Fig, MDDLogo applies the chi-square test χ^2^(A_i_, A_j_) to iteratively evaluate the dependence of the occurrence of amino acids in two positions, *A*_*i*_ and *A*_*j*_, which are neighboring to the substrate site. Based on the biochemical properties of amino acids, the 20 amino acids were categorized into 5 groups: the polar, acidic, basic, hydrophobic, and aromatic groups (S1 Table). A contingency table describes the frequency of existence of twenty amino acids in positions *A*_*i*_ and *A*_*j*_. The chi-square test was defined as:

$$\chi^{\text{2}}{\text{(A}}_{\text{i}}{,\text{A}}_{\text{j}}\text{)}=\sum_{m=1}^{5}\sum_{n=1}^{5}\frac{{\left(X_{mn}-E_{mn}\right)}^{2}}{E_{mn}}$$ (1)

where *X*_*mn*_ is the number of sequences that had amino acids of group *m* in position *A*_*i*_ and amino acids of group *n* in position *A*_*j*_, for each pair (*A*_*i*_, *A*_*j*_) with *i* ≠ *j*. *E*_*mn*_ was measured as $\frac{X_{mR}\cdot X_{Cn}}{X}$, where *X*_*mR*_ = *X*_*m1*_ + …+ *X*_*m5*_, *X*_*Cn*_ = *X*_*1n*_ + …+ *X*_*5n*_ and *X* represents the total number of sequences. If a strong dependence was discovered (described as a *X*^*2*^ value greater than 34.3, proportional to a cutoff level of *P* = 0.01 with 16 degrees of freedom) between two positions, it followed the description of Burge and Karlin [44]. After the recursive chi-square test, MDDLogo divides a group of aligned sequences into subsets that capture the most significant dependencies of positions on each other. When applying MDDLogo, a parameter, i.e., the maximum cluster size, should be set. If the size of a subgroup is less than the specified value of maximum cluster size, the subgroup will not be divided any further. MDDLogo will be terminated when all the subgroup sizes are less than the specified value of maximum cluster size [23].

### Construction of predictive model

In this study, we employed a support vector machine to build predictive models for discriminating *S*-palmitoylation sites and *non*-*S*-palmitoylation sites in a training dataset. Based on a binary classification, a kernel function transformed the input samples into a higher dimensional space and then found a hyper-plane to discriminate between the two classes with maximal margin and minimal error. This study employed a public SVM library (LIBSVM) [56] to implement the predictive model for distinguishing *S*-palmitoylation sites from non- *S*-palmitoylation sites. The radial basis function (RBF):

$${\text{K(S}}_{\text{i}}{,\text{S}}_{\text{j}}\text{)}=\text{exp}(-\gamma {\left\|S_{i}-S_{j}\right\|}^{2})$$ (2)

was adopted as the kernel function for learning in the SVM classifier. Two supporting factors that enhance performance are gamma and cost. The RBF kernel is determined by the gamma parameter, while the cost parameter controls the hyper-plane softness.

**Fig 1:**
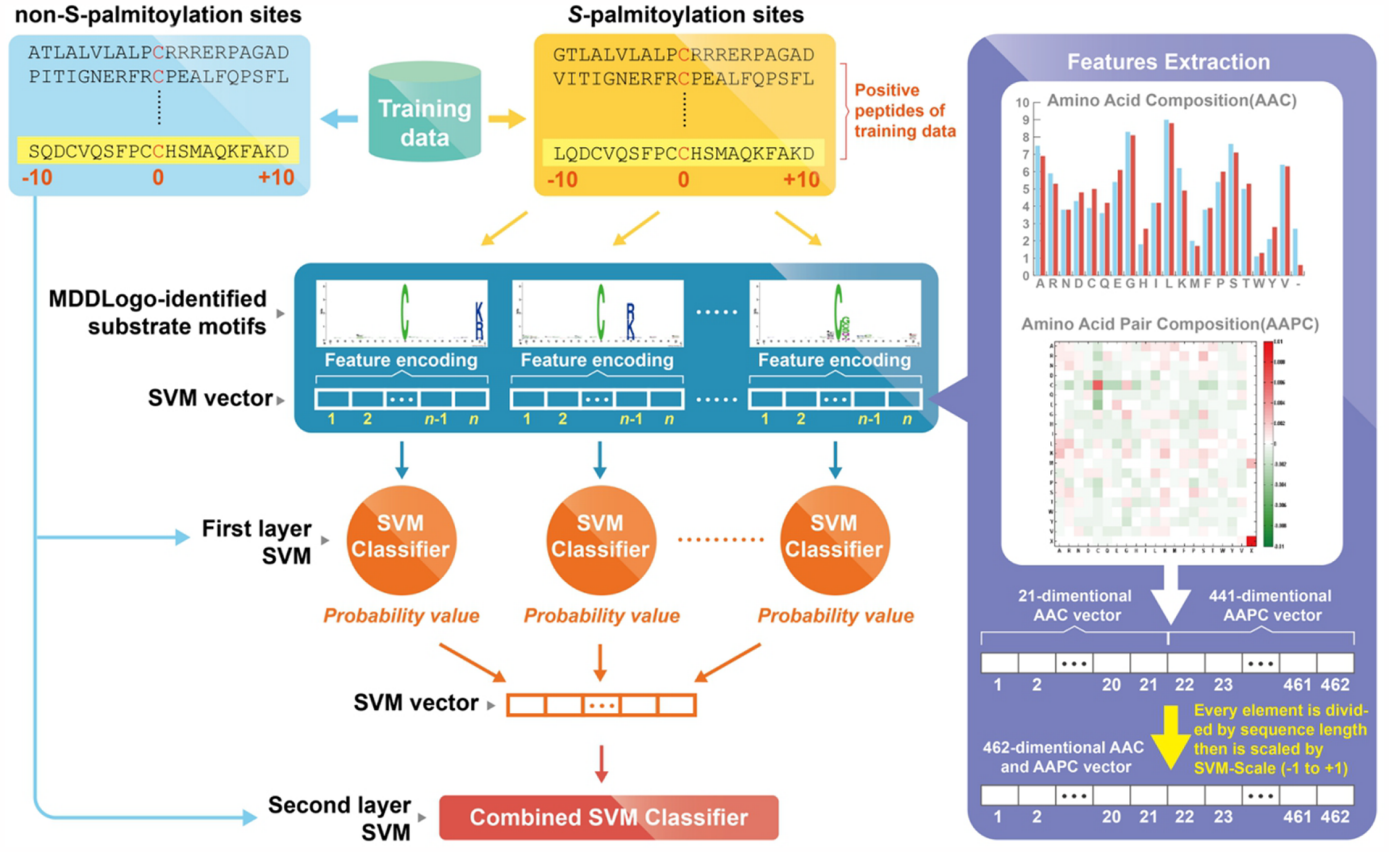
The conceptual diagram of constructing two-layered SVMs based on MDDLogo-identified substrate motifs.

In this study, each feature was used to generate a predictive model based on the LIBSVM library; then, the best feature was selected as the training feature to construct a predictive model for each MDDLogo-clustered subgroup. As shown in Fig 1, LIBSVM was employed to generate a first-layered SVM model for each MDDLogo-identified substrate motif. The negative data for each MDDLogo-clustered subgroup were selected from the negative training dataset (5676 non-*S*-palmitoylated sequences) in a ratio of approximately 1:8 (which approximates the ratio of the number of positive data to the number of negative data, 710:5676). In first layer, each SVM model outputs a probability estimate ranging from 0 to 1 for each prediction. Thus, the probability estimates from each SVM classifier trained according to a specific motif were adopted to form an input vector for the second-layered SVM classifier.

### Evaluation of predictive performance

To determine the best model, five-fold cross-validation was carried out for models trained with each different feature in order to evaluate their predictive performances. The training data were divided into five approximately equal subgroups. The ratio of test and training sets was 1:4, and the cross-validation process was run five times. The five validation results were then combined to generate a single estimation. Cross-validation evaluation improves the reliability of evaluation, because it considers all original data, in both the training and testing data sets, in general, and tests each subset only once [57]. To gauge the effective predictive performance of training model, the following measures were used: sensitivity (Sn), specificity (Sp), accuracy (Acc) and Matthews Correlation Coefficient (MCC):

$$Sn=\frac{TP}{TP+FN}$$ (3)

$$Sp=\frac{TN}{TN+FP}$$ (4)

$$Acc=\frac{TP+TN}{TP+FN+TN+FP}$$ (5)

$$MCC=\frac{\left(TP\times TN)-(FN\times FP\right)}{\sqrt{\left(TP+FN)\times (TN+FP)\times (TP+FP)\times (TN+FN\right)}}$$ (6)

where TP, TN, FP and FN represent the numbers of true positives, true negatives, false positives and false negatives, respectively. Sensitivity is the percentage of correct predictions from positive data (*S*-palmitoylated cysteines), while specificity represents that from negative data (non- *S*-palmitoylated cysteines). Accuracy reflects the overall proportion of correctly predicted positive data and negative data. For binary classifications, accuracy is sometimes not useful when the two classes are of very different sizes [32]. Therefore, the MCC is typically considered as a balanced measure, even if the two classes are of very different sizes [58]. The MCC value ranges from −1 to +1, while the values of other three measures range from 0 to 1. A coefficient value of +1 represents a perfect prediction, while the values 0 and −1 represent random and opposite predictions, respectively. A higher positive MCC value indicates a better prediction for correctly classifying positive and negative data. Moreover, the ROC (Receiver Operating Characteristic) curves of various models are measured for the comparison of their predictive performances and stabilities. Finally, after selecting the best predictive model with the highest MCC in this study, an independent test was carried out on the final model with the best performance in cross-validation evaluation.

## Results and discussion

### Investigation of amino acid composition surrounding *S*-palmitoylation sites

**Fig 2:**
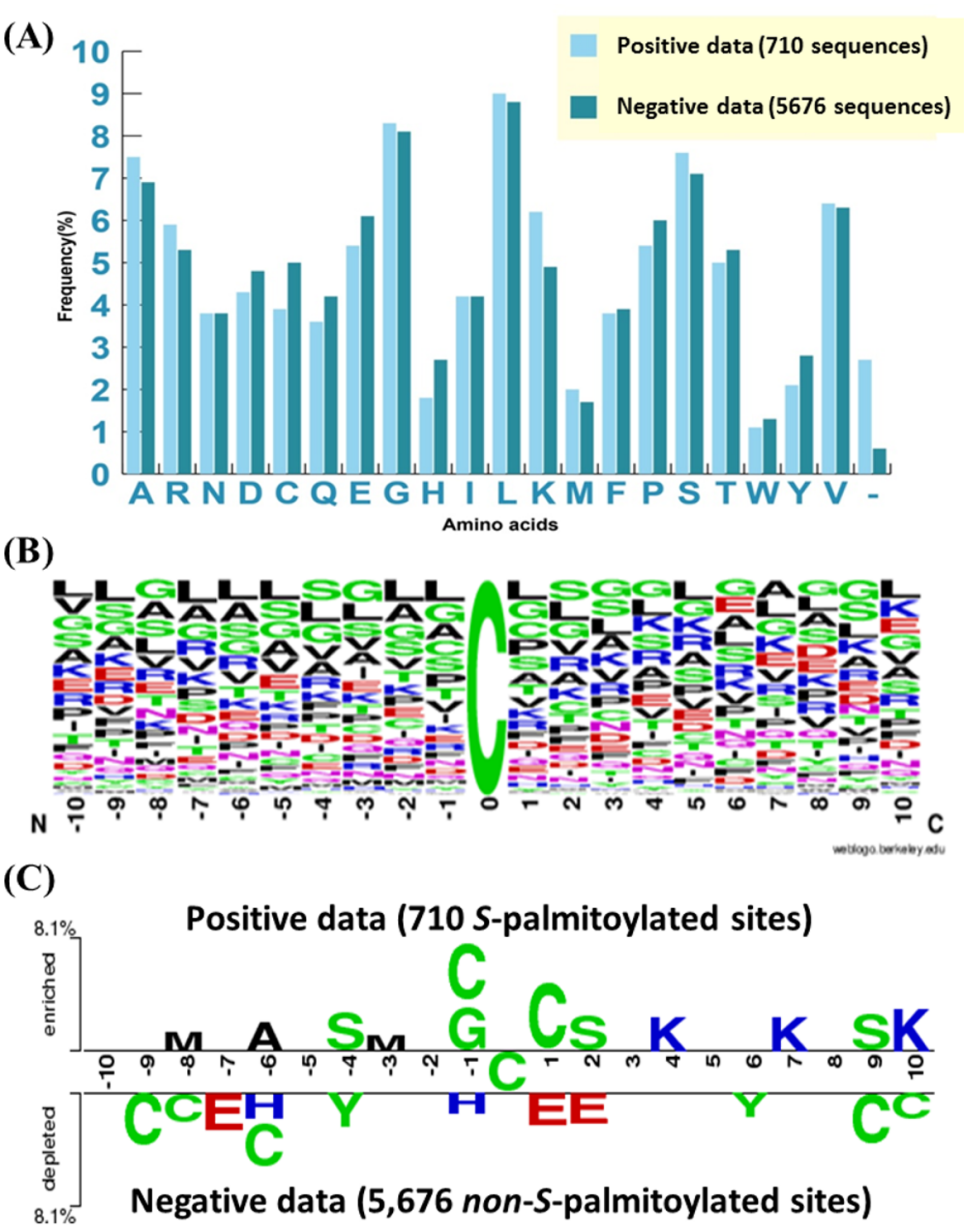
Amino acids composition of the *S*-palmitoylation sites. (A) Comparison of amino acids composition between positive data (710 *S*-palmitoylation sites) and negative data (5,676 *non*-*S*-palmitoylation sites). (B) Position-specific amino acids composition surrounding the *S*-palmitoylation sites based on frequency plot of WebLogo. (C) The compositional biases of amino acids around *S*-palmitoylation sites (upper panel) compared to the *non*-*S*-palmitoylation sites (lower panel) based on TwoSampleLogo (*p*-value < 0.01).

The frequency of occurrence of 20 amino acids surrounding *S*-palmitoylation sites was investigated based on 710 fragment sequences with 21-mer window length to explore the potential consensus motifs. Fig 2A indicates that at *S*-palmitoylation sites, lysine (K) residue occurs at a higher frequency, while cysteine (C), glutamic acid (E) and histidine (H) residues have a lower frequency of occurrence. Additionally, WebLogo [59] was utilized to compute the position-specific amino acid composition for *S*-palmitoylation (Fig 2B). However, it is difficult to compare the amino acid composition of *S*-palmitoylation and non-*S*-palmitoylation sites at a specific position. Thus, Two Sample Logo [60] was used to detect differences in position-specific symbol compositions between the *S*-palmitoylated and non-*S*-palmitoylated datasets. Cysteine was placed in the middle of the fragment sequences, and positions of the flanking amino acids ranged from −10 to +10. The comparison of 710 *S*-palmitoylated sites and 5676 non-*S*-palmitoylated sites in Fig 2C indicates that the positively charged amino acids, such as lysine (K) residues, had the highest ratios at positions +4, +7, and +10 (with *P* < 0.01). It also shows a slight abundance of polar amino acids such as glycine (G), cysteine (C), and serine (S) at positions -4, -1, +1, +2, and +9. Position −1 was a special case, exhibiting the highest proportion of the polar group of residues; namely glycine (G) and cysteine (C). By contrast, the negatively charged E residue was depleted at positions −7, +1, and +2. This analysis shows that the distance among amino acid characteristics in a sequence plays a vital role in distinguishing between *S*-palmitoylated and non-*S*-palmitoylated sites.

### Selection of the best feature based on five-fold cross-validation

To determine the best feature for discriminating between *S*-palmitoylation sites and non-*S*-palmitoylation sites, SVM models were built using various features, including AA, AAC, AAPC, PWM, PSSM, BLOSUM62, and ASA. Each predictive model was evaluated based on four measures—sensitivity (Sn), specificity (Sp), accuracy (Acc), and Matthews correlation coefficient (MCC)—using five-fold cross-validation. As shown in Table 2, the SVM model trained with AAC had the highest MCC value at 0.29, and relatively high sensitivity, specificity, and accuracy at 0.68, 0.69, and 0.69, respectively. The SVM model trained using AAPC with a 441-dimensional vector yielded a comparable performance, with a sensitivity of 0.67, specificity of 0.68, accuracy of 0.68, and MCC value of 0.26. The SVM model, trained using PSSM with a 420-dimensional vector, also performed as effectively as the AAPC model. On the other hand, the accessible surface area (ASA) was found to be the worst feature for the prediction of *S*-palmitoylation sites, with a sensitivity of 0.56, specificity of 0.57, accuracy of 0.57, and MCC of 0.09. Along with the performance evaluation of single features, the AAC feature with the best performance was combined with other features in order to obtain better predictive power. As provided in Table 2, features B62, AAPC, and PSSM were found to combine well with AAC in terms of the improvement of predictive performance. Overall, the SVM model based on a hybrid of AAC and PSSM features provided the best predictive performance in Sn, Sp, Acc, and MCC at 0.72, 0.73, 0.73, and 0.38, respectively. Therefore, the hybrid feature of AAC and PSSM was selected as the best feature for the construction of predictive models.

**Table 2:** Five-fold cross validation results on single SVM model trained with various features. Sn, sensitivity; Sp, specificity; Acc, accuracy; MCC, Matthews Correlation Coefficient; AUC, area under the curve of ROC.

Training features	Sn	Sp	Acc	MCC	AUC
20D Binary code (AA)	0.60	0.62	0.62	0.16	0.61
BLOSUM62 (B62)	0.62	0.63	0.63	0.18	0.62
Amino Acid Composition (AAC)	0.68	0.69	0.69	0.29	0.70
Amino Acid Pair Composition (AAPC)	0.67	0.68	0.68	0.26	0.68
Accessible Surface Area (ASA)	0.56	0.57	0.57	0.09	0.58
Position Weight Matrix (PWM)	0.65	0.66	0.66	0.22	0.66
Position-specific scoring matrix (PSSM)	0.67	0.68	0.68	0.26	0.68
AAC + AA	0.68	0.69	0.69	0.29	0.70
AAC + B62	0.67	0.70	0.70	0.31	0.73
AAC + AAPC	0.71	0.71	0.71	0.34	0.76
AAC + ASA	0.66	0.68	0.68	0.24	0.67
AAC + PWM	0.70	0.70	0.70	0.32	0.75
AAC + PSSM	0.72	0.73	0.73	0.38	0.78

**Fig 3:**
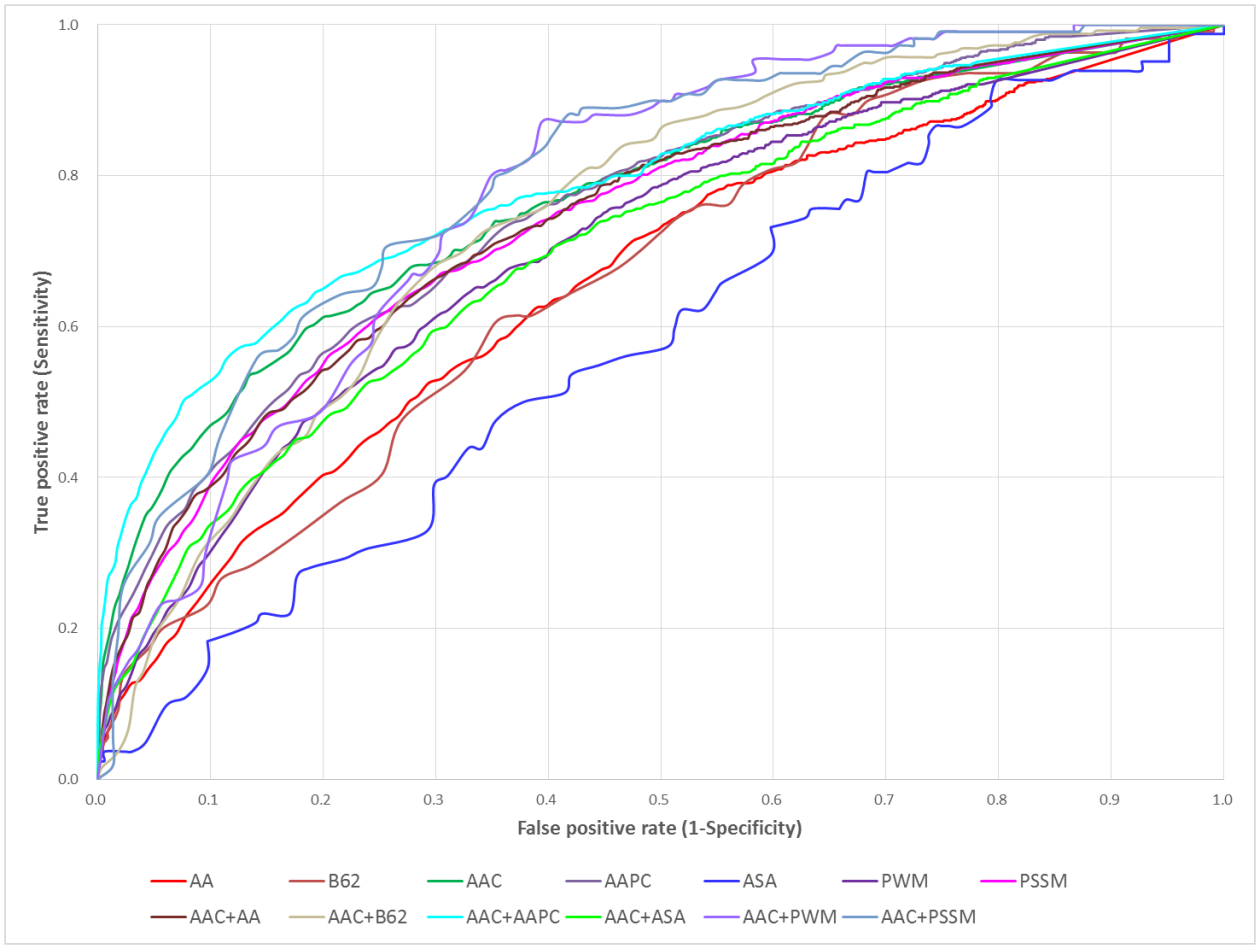
ROC curves of the single SVM models trained using various features based on five-fold cross-validation.

Based on the evaluation of five-fold cross-validation, Fig 3 presents the comparison of ROC curves between the predictive models trained using various features; additionally, the values of AUC (Area Under the Curve of ROC) are provided in Table 2, which also indicated that the SVM model trained using a hybrid of AAC and PSSM features could obtain the best prediction outcome with an AUC value of 0.78. In order to examine the predictive robustness of the selected SVM model, the four-, six-, eight-, and ten-fold cross validation results also have been provided in S2, S3, S4 and S5 Tables, respectively. Furthermore, the comparison of ROC curves based on four-, six-, eight- and ten-fold cross validation are presented in S3, S4, S5 and S6 Figs, respectively. According to a variety of evaluation criteria, the SVM model trained using a hybrid of AAC and PSSM features could perform an overall best performance among various predictive models.

### MDD-identified motif signatures for *S*-palmitoylation sites

**Fig 4:**
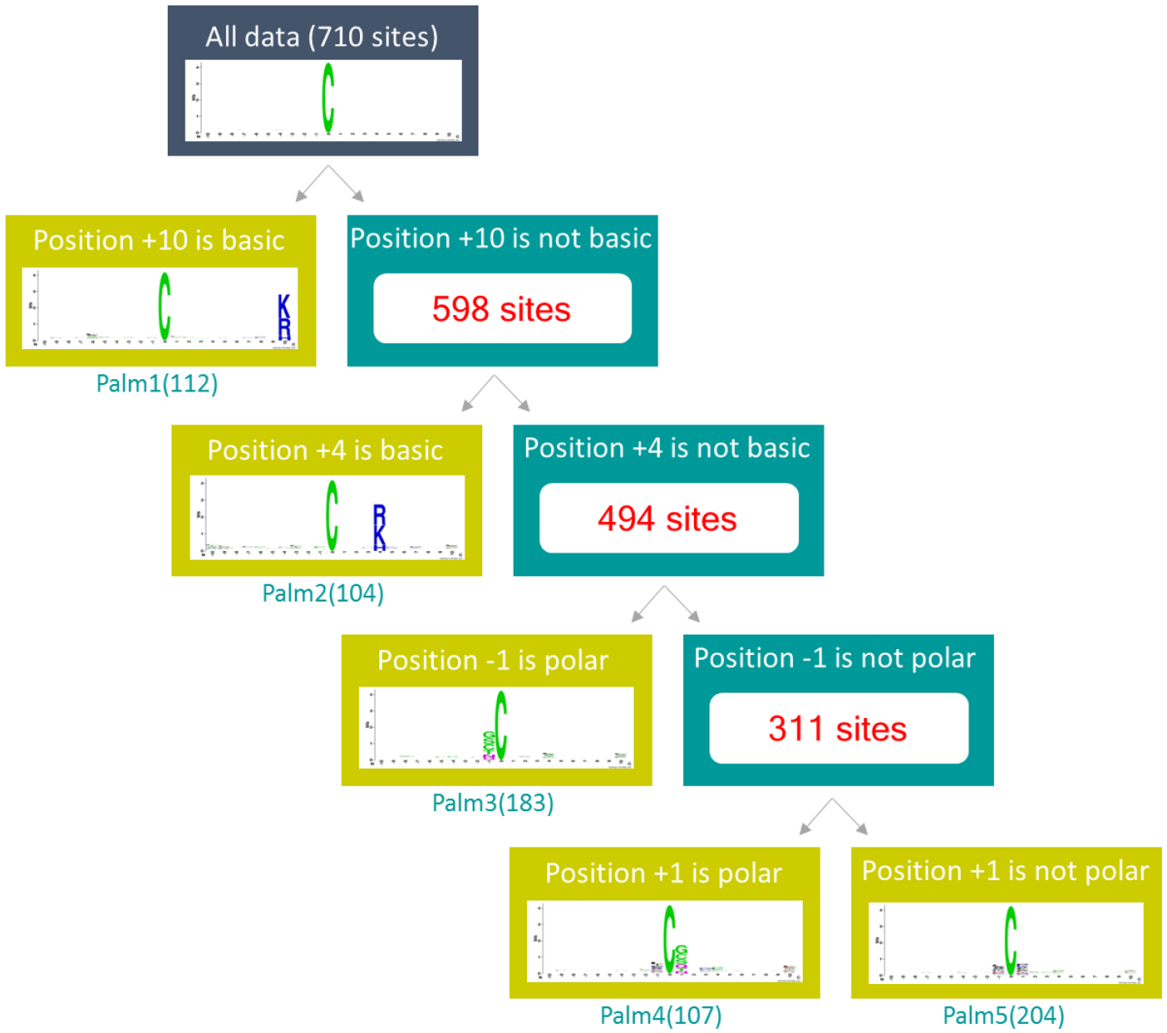
Tree-like view of MDDLogo-identified motif signatures on 710 non-homologous *S*-palmitoylated sequences.

In this investigation, MDDLogo was utilized to explore the motif signatures by dividing the positive training dataset (710 sites) into five subgroups. Each subgroup represents a potential substrate specificity that contains statistically significant dependencies of amino acid composition in specific positions. Fig 4 provides a tree-like visualization of MDDLogo-clustered subgroups with statistically significant motifs for 710 non-homologous *S*-palmitoylation sites. On the left subtree, one motif (subgroup Palm1) out of all the MDDLogo-clustered subgroups was detected based on the occurrence of basic amino acids (K, R, and H) at position +10, with maximal dependence value. At the same time, the remaining dataset (598 sites) was further examined for the maximal dependence of occurrence of amino acids at other positions. Subgroup Palm2 (104 sites) had a similar motif of basic amino acids at position +4. This result was consistent with a previous study [9]: palmitoylated cysteine is surrounded by basic or hydrophobic amino acids. Additionally, subgroups Palm3 (183 sites) and Palm4 (107 sites) had polar amino acids at positions -1 and +1, respectively. Finally, the remaining 204 positive sequences resulted in the fifth subgroup (Palm5), which contained a slight conservation of amino acids at positions -1 and +1.

### Effectiveness of incorporating MDD-identified motifs into the identification of *S*-palmitoylation sites

**Fig 5:**
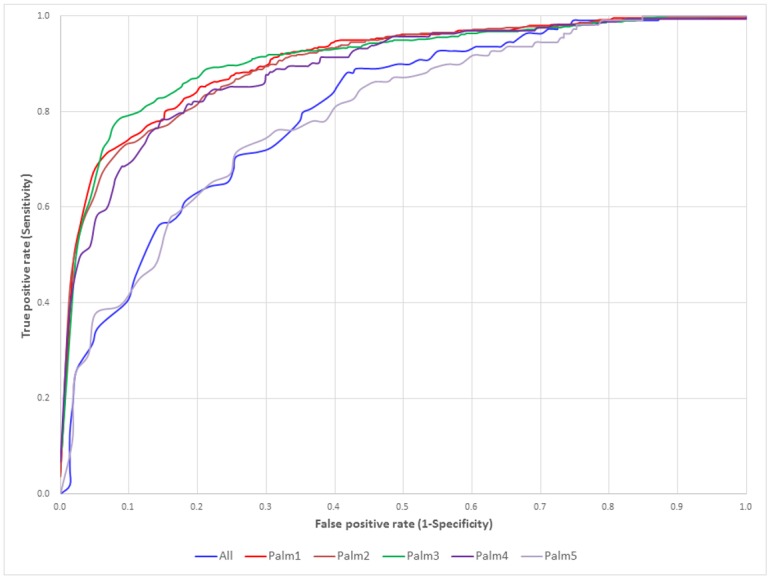
ROC curves of the SVM models trained from MDDLogo-identified motifs based on five-fold cross-validation.

In order to evaluate the predictive power of MDDLogo-identified substrate motifs in discriminating between *S*-palmitoylation sites and non-*S*-palmitoylation sites, LIBSVM was utilized to generate a predictive model for each subgroup based on the best hybrid feature, AAC combined with PSSM. Based on the evaluation of five-fold cross-validation, Fig 5 provides the comparison of ROC curves between the SVM models trained using all dataset and MDDLogo-clustered subgroups. In addition, Table 3 provides the predictive sensitivity, specificity, accuracy, and MCC for each subgroup, based on their five-fold cross-validation performances. The values of ROC are also given in Table 3. It shows that subgroup Palm3 with G/S/T/C/Q/N motif at position -1 had the highest performance for sensitivity, specificity, accuracy, MCC, and AUC values at 0.83, 0.84, 0.84, 0.53, and 0.89, respectively. Subgroup Palm1 had predictive powers of sensitivity, specificity, accuracy, MCC, and AUC of 0.82, 0.83, 0.83, 0.50, and 0.87, respectively, which is comparable to those of subgroup Palm3. Subgroup Palm5 with a slightly conserved motif generally showed relatively low performance, with a sensitivity of 0.73, specificity of 0.73, accuracy of 0.73, MCC of 0.39, and AUC value of 0.79. Overall, the five subgroups, containing the conserved motif of amino acids at specific positions, yielded promising accuracy as well as balanced sensitivity and specificity. In order to incorporate the five motifs into the identification of *S*-palmitoylation sites with substrate specificity, the five SVM models trained from MDDLogo-clustered subgroups were incorporated into a two-layered SVM model. The values of probability estimated from five SVM models according to a specific motif signature were combined to form an input vector for the second-layered SVM classifier. As shown in Table 3, based on an evaluation of five-fold cross-validation, the predictive performance of the two-layered SVM model was significantly improved as compared to the single SVM model trained from all datasets without MDD clustering. The two-layered SVM model provided sensitivity, specificity, accuracy, MCC, and AUC values of 0.79, 0.80, 0.80, 0.45, and 0.85, respectively. In summary, the two-layered SVM model combining all MDDLogo-identified motif signatures can be expected to enhance performance, and could be implemented as a web-based prediction resource.

**Table 3:** Five-fold cross-validation performance for five SVM models trained from MDDLogo-identified motifs.

Dataset	Number of positive data	Number of negative data	Sn	Sp	Acc	MCC	AUC
All data	710	5,676	0.72	0.73	0.73	0.38	0.78
Palm1	112	895	0.82	0.83	0.83	0.50	0.87
Palm2	104	831	0.81	0.81	0.81	0.48	0.86
Palm3	183	1463	0.83	0.84	0.84	0.53	0.89
Palm4	107	856	0.79	0.81	0.81	0.47	0.86
Palm5	204	1631	0.73	0.73	0.73	0.39	0.79
**Combined result**	**710**	**5676**	**0.79**	**0.80**	**0.80**	**0.45**	**0.85**

### Independent testing and comparison with existing prediction tools

An independent test set of *S*-palmitoylation sites in mouse taken from Gould et al. [30] consisting of 613 positive sites and 5412 negative sites was used to further compare the predictive power of the single SVM model trained using all training data and the two-layered SVM model trained using the five MDDLogo-identified motifs (S7 Fig). As shown in Table 4, the single SVM model yielded a sensitivity of 0.584, a specificity of 0.702, an accuracy of 0.690, and an MCC of 0.184. Meanwhile, the performance of the two-layered SVM model achieved a sensitivity of 0.690, a specificity of 0.694, an accuracy of 0.693, and an MCC of 0.244. To further demonstrate the effectiveness of our predictive model, the independent test set was utilized to compare the two-layered SVM model with existing prediction tools. Because several predictors are not available online, the comparison was carried out using CSS-Palm 4.0 [16,17], NBA-Palm [18], CKSAAP-Palm [19], WAP-Palm [21], PalmPred [22], and SeqPalm [61] based on an independent testing dataset. As shown in Table 4, this independent testing indicated that most of the predictors have relatively high accuracy; however, they provide an unbalanced prediction performance: higher specificity accompanied with lower sensitivity. Overall, the testing results revealed that our method could provide balanced sensitivity and specificity in the prediction of *S*-palmitoylation sites. Additionally, the comparison of ROC curves between our method and other prediction tools were provided in S8 Fig. Without the complete information of predicted results from other prediction tools, the ROC curves could not be illustrated completely for several prediction tools. However, the comparison of ROC curves showed that the proposed method could outperform other prediction methods at a specified level of false positive rate (1—specificity).

**Table 4:** Comparison of independent testing results between our methods and other *S*-palmitoylation prediction tools.

Methods	TP	FN	TN	FP	Sn	Sp	Acc	MCC
Single SVM	358	255	3801	1611	0.584	0.702	0.690	0.184
Two-Layered SVM	423	190	3755	1657	0.690	0.694	0.693	0.244
SeqPalm	22	591	5141	271	0.036	0.950	0.857	-0.020
CSKAAP-Palm	43	570	5102	310	0.070	0.943	0.854	0.017
CSS-Palm 4.0	209	404	4817	595	0.341	0.890	0.834	0.205
NBA-Palm	19	594	4673	739	0.031	0.863	0.778	-0.096
WAP-Palm	102	511	4711	701	0.167	0.870	0.798	0.033
PalmPred	169	444	4474	938	0.276	0.827	0.771	0.080

### Web-based system for the identification of *S*-palmitoylation sites

**Fig 6:**
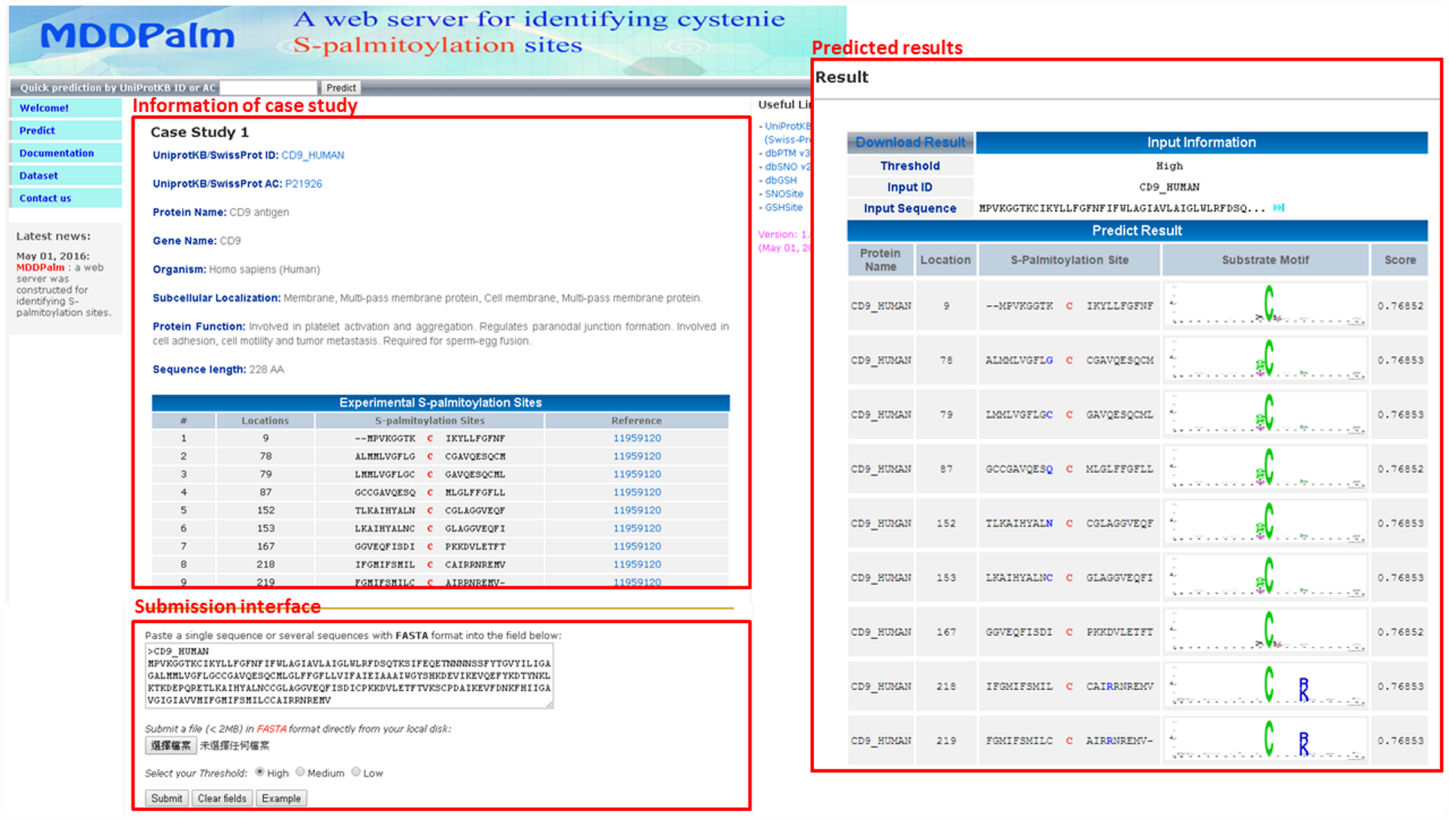
A case study of *S*-palmitoylation site prediction on human CD9 antigen (CD9_HUMAN).

Owing to the time-consuming and labor-intensive process of experimentation, constructing an effective prediction system can aid in the study of *S*-palmitoylation sites. Based on cross-validation evaluation and independent testing, the two-layered SVM model combining all MDDLogo-identified substrate motifs and based on the hybrid features of AAC and PSSM was used in the construction of a web-based prediction system called MDD-Palm. After users submit their protein sequences in FASTA format, MDD-Palm returns the predicted results containing *S*-palmitoylation sites, the flanking amino acids, and the corresponding MDDLogo-identified motifs. Human CD9 antigen (CD9_HUMAN) was utilized to demonstrate the effectiveness of MDD-Palm. The human CD9 antigen contains nine verified *S*-palmitoylation sites at Cys-9, Cys-78, Cys-79, Cys-87, Cys-152, Cys-153, Cys-167, Cys-218, and Cys-219 [62]. As shown in Fig 6, MDD-Palm produced accurate positive predictions at all of the previously identified *S*-palmitoylation sites, according to the corresponding motif signatures.

## Conclusion

In this study, we proposed a bioinformatics method for the characterization and identification of *S*-palmitoylation sites with substrate site specificity. Two Sample Logo revealed that the most pronounced feature of *S*-palmitoylation sites is the enrichment of the positively charged amino acids (K, R, and H) at specific positions as well as the polar amino acids at positions -1 and +1. According to evaluation by five-fold cross-validation, the SVM model trained with a hybrid combination of AAC and PSSM features achieved the highest sensitivity, specificity, accuracy, and MCC. As stated previously, the main purpose of this study was to explore the substrate motifs of *S*-palmitoylation sites based on amino acid sequences. Using MDDLogo, the *S*-palmitoylated sequences were clustered into five subgroups corresponding with five motif signatures. The MDDLogo-identified motifs can thus be used to construct a two-layered SVM model to significantly enhance the predictive performance of the *S*-palmitoylation site. An independent testing dataset was used to evaluate the two models: the two-layered SVM model and single SVM model without MDD. As expected, the two-layered SVM model, which combined MDDLogo-identified motifs, achieved a better predictive performance. Consequently, this model was employed to build a web-based resource called MDD-Palm to identify *S*-palmitoylation sites and their corresponding substrate motifs.

## Supporting information

S1 Fig The encoding scheme of the position-specific scoring matrix (PSSM) combined with BLOSUM62 feature. (TIF)

S2 Fig The analytical flowchart of MDDLogo application. (JPG)

S3 Fig ROC curves of the single SVM models trained using various features based on four-fold cross-validation. (TIF)

S4 Fig ROC curves of the single SVM models trained using various features based on six-fold cross-validation. (TIF)

S5 Fig ROC curves of the single SVM models trained using various features based on eight-fold cross-validation. (TIF)

S6 Fig ROC curves of the single SVM models trained using various features based on ten-fold cross-validation. (TIF)

S7 Fig Comparison of independent testing performance between single SVM model and two-layered SVM model. (TIF)

S8 Fig Comparison of ROC curves between our methods and other *S*-palmitoylation prediction tools based on independent testing results. (TIF)

S1 Table The amino acids group of MDDLogo used in this study. (DOCX)

S2 Table Four-fold cross validation results on single SVM model trained with various features. (DOCX)

S3 Table Six-fold cross validation results on single SVM model trained with various features. (DOCX)

S4 Table Eight-fold cross validation results on single SVM model trained with various features. (DOCX)

S5 Table Ten-fold cross validation results on single SVM model trained with various features. (DOCX)
