# Supplementary figures and images for: MDD-Palm: Identification of protein S-palmitoylation sites with substrate motifs based on maximal dependence decomposition
Source: PLoS One. 2017 Jun 29;12(6):e0179529. doi: 10.1371/journal.pone.0179529 (PMC5491019; doi:10.1371/journal.pone.0179529)

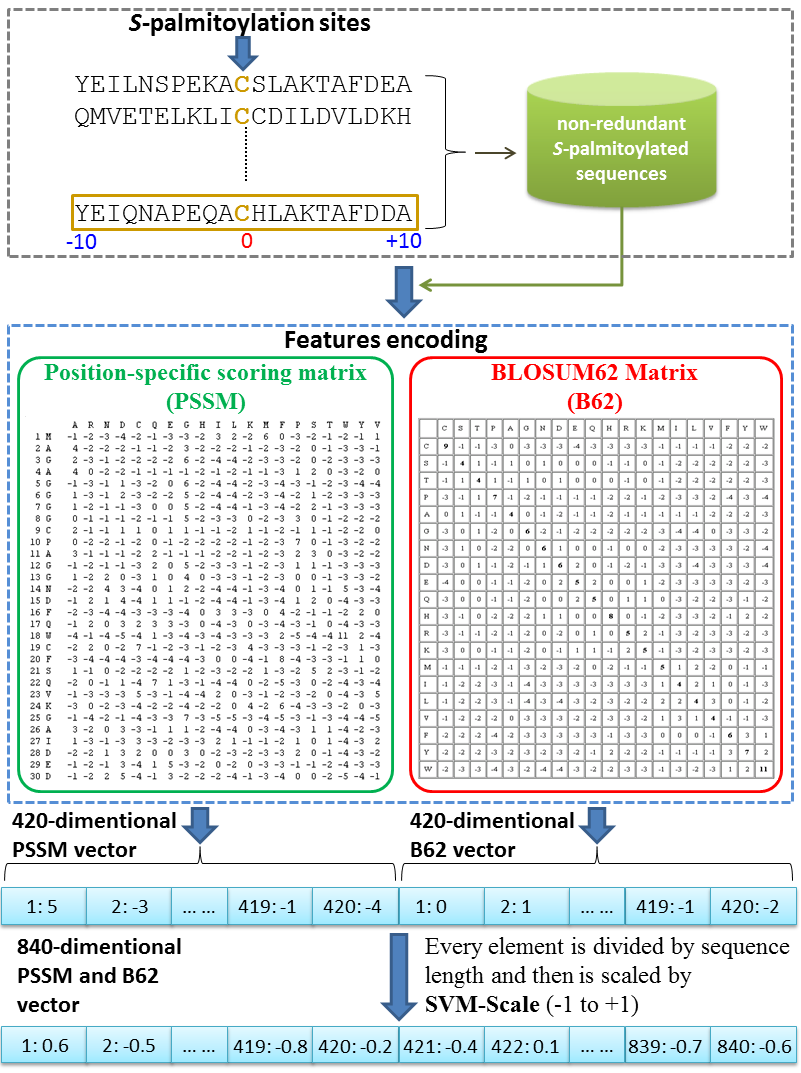

Supplement: S1 Fig — (TIF) [file pone.0179529.s001.tif]

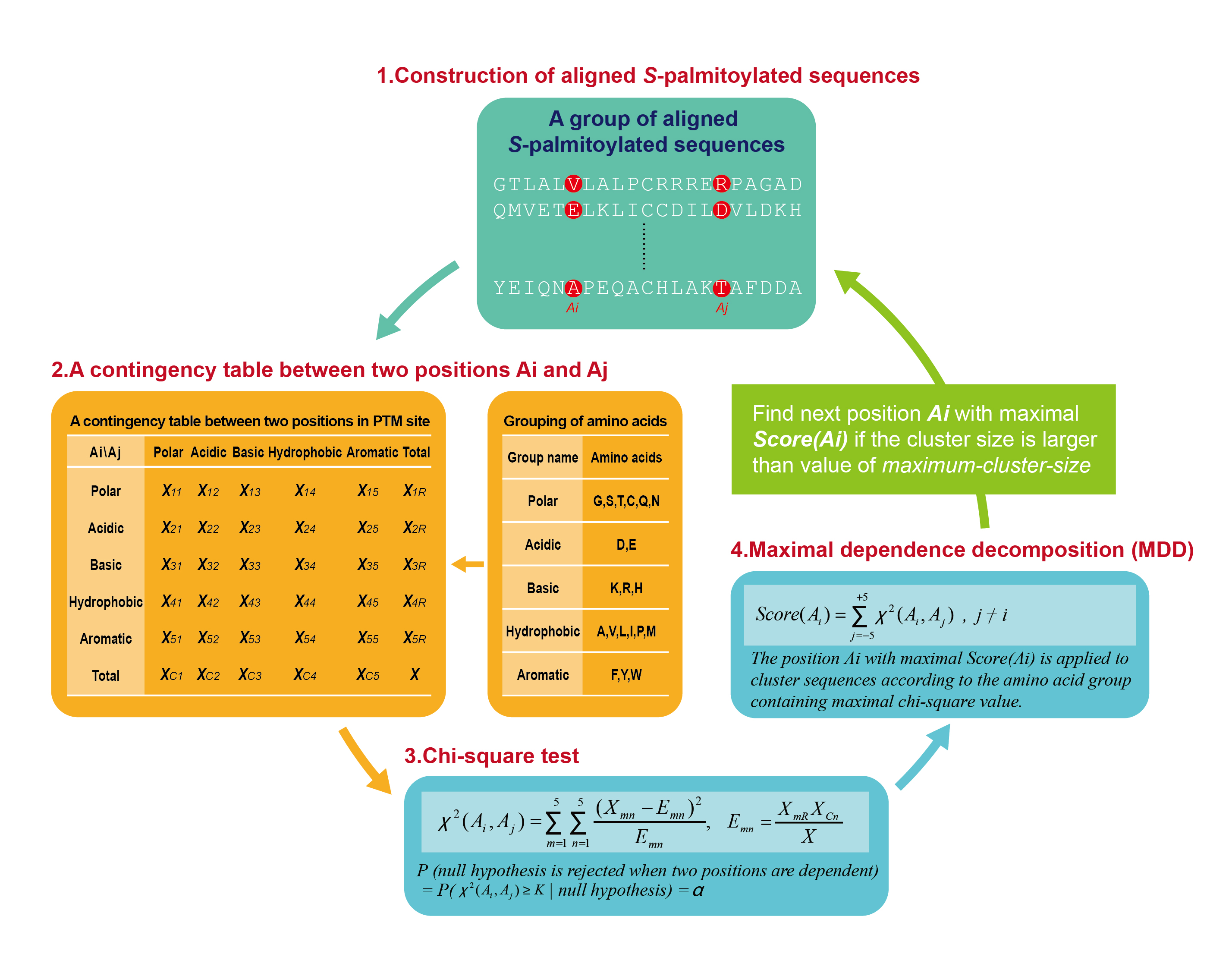

Supplement: S2 Fig — (JPG) [file pone.0179529.s002.jpg]

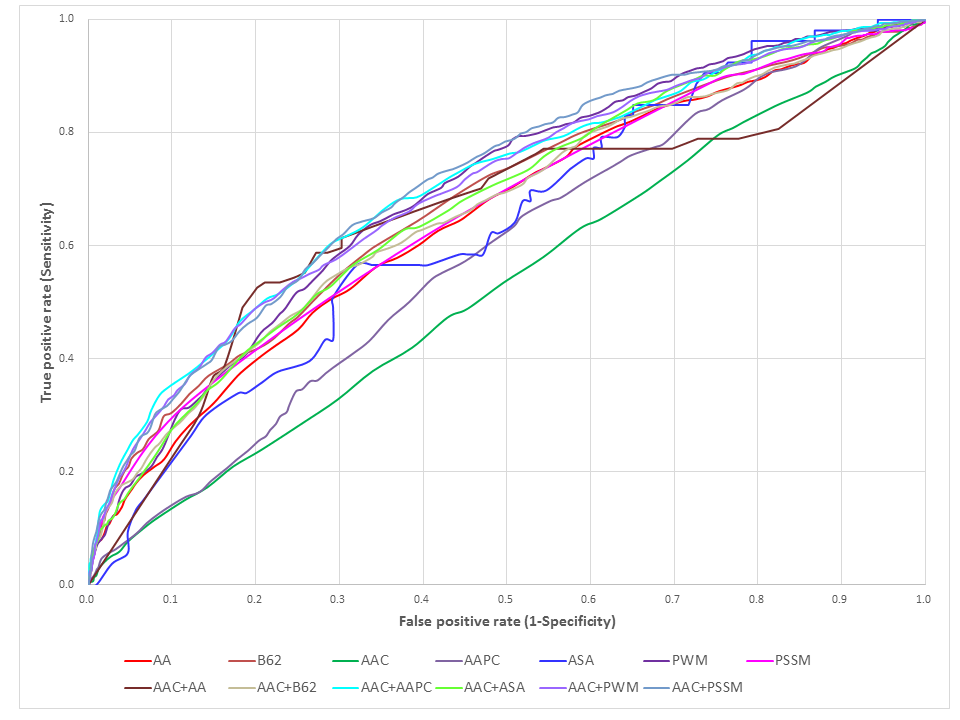

Supplement: S3 Fig — (TIF) [file pone.0179529.s003.tif]

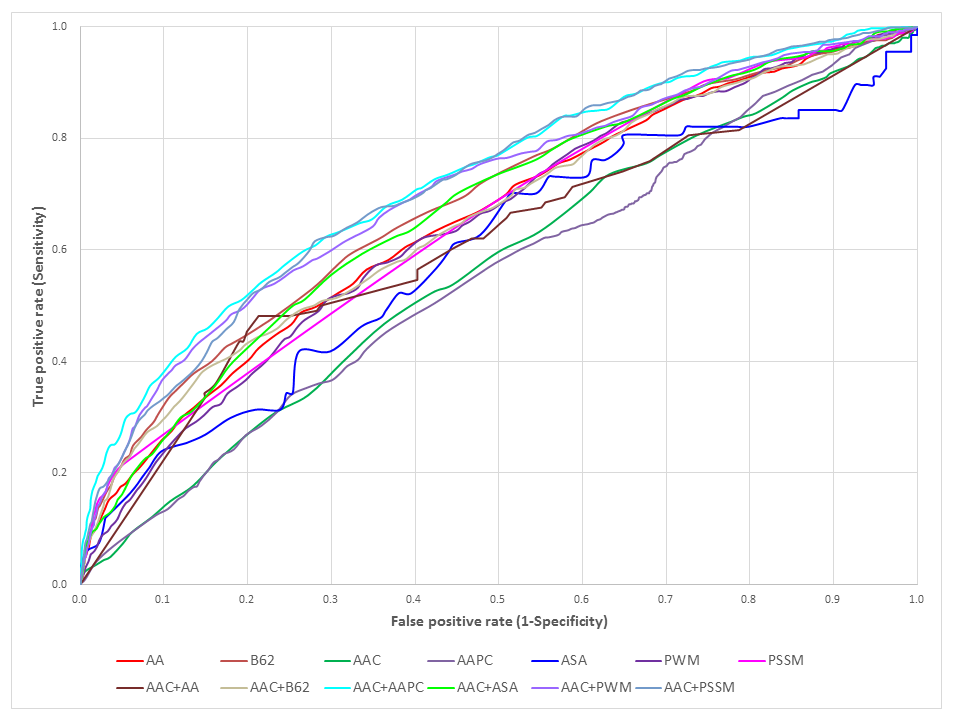

Supplement: S4 Fig — (TIF) [file pone.0179529.s004.tif]

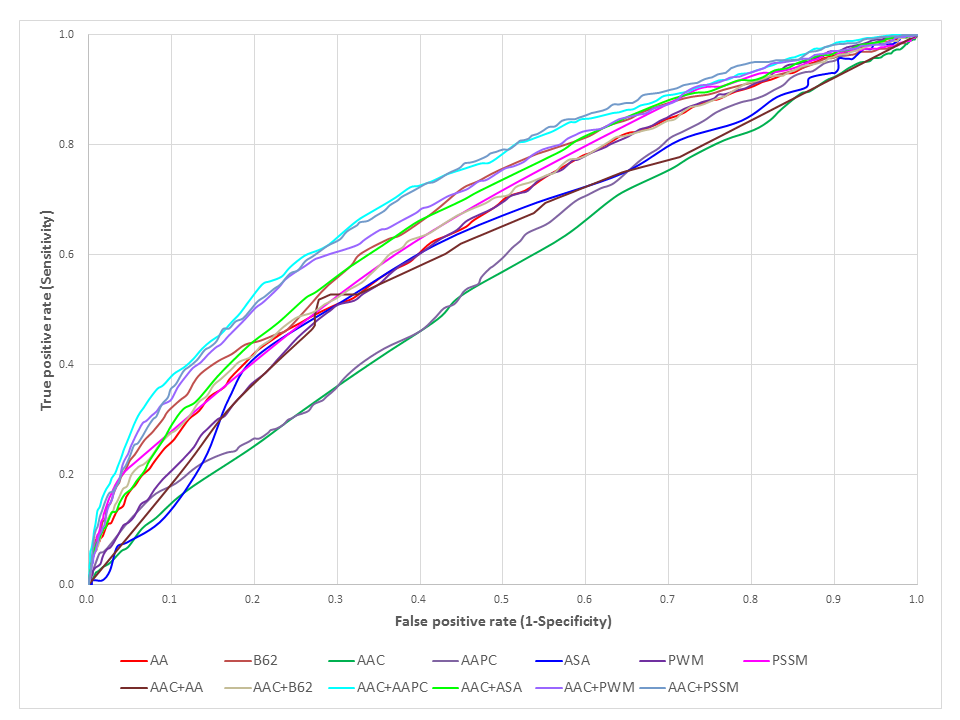

Supplement: S5 Fig — (TIF) [file pone.0179529.s005.tif]

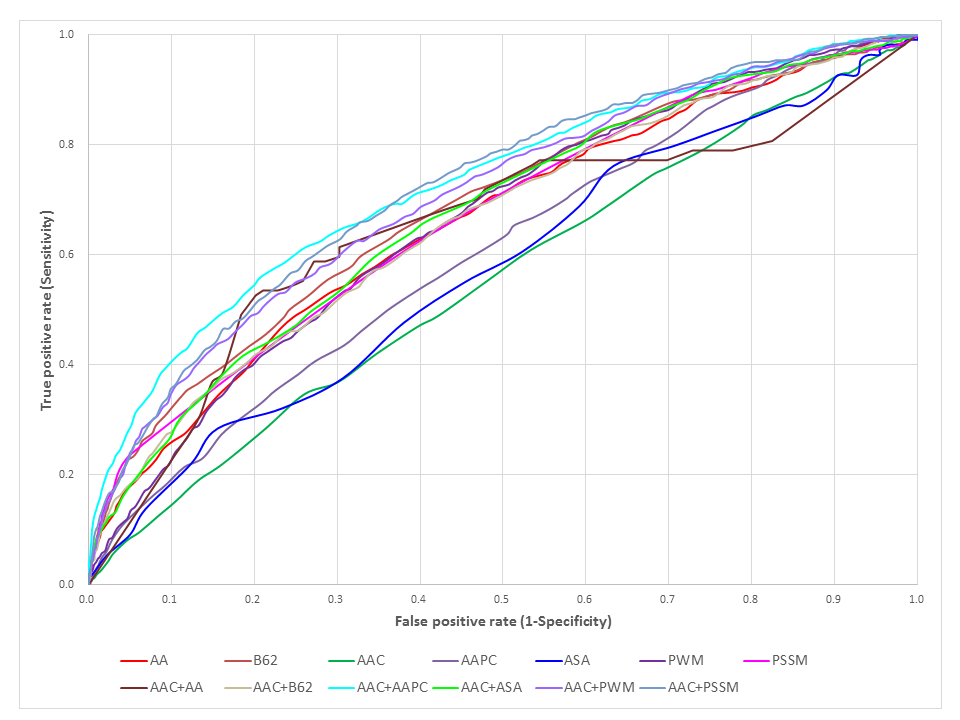

Supplement: S6 Fig — (TIF) [file pone.0179529.s006.tif]

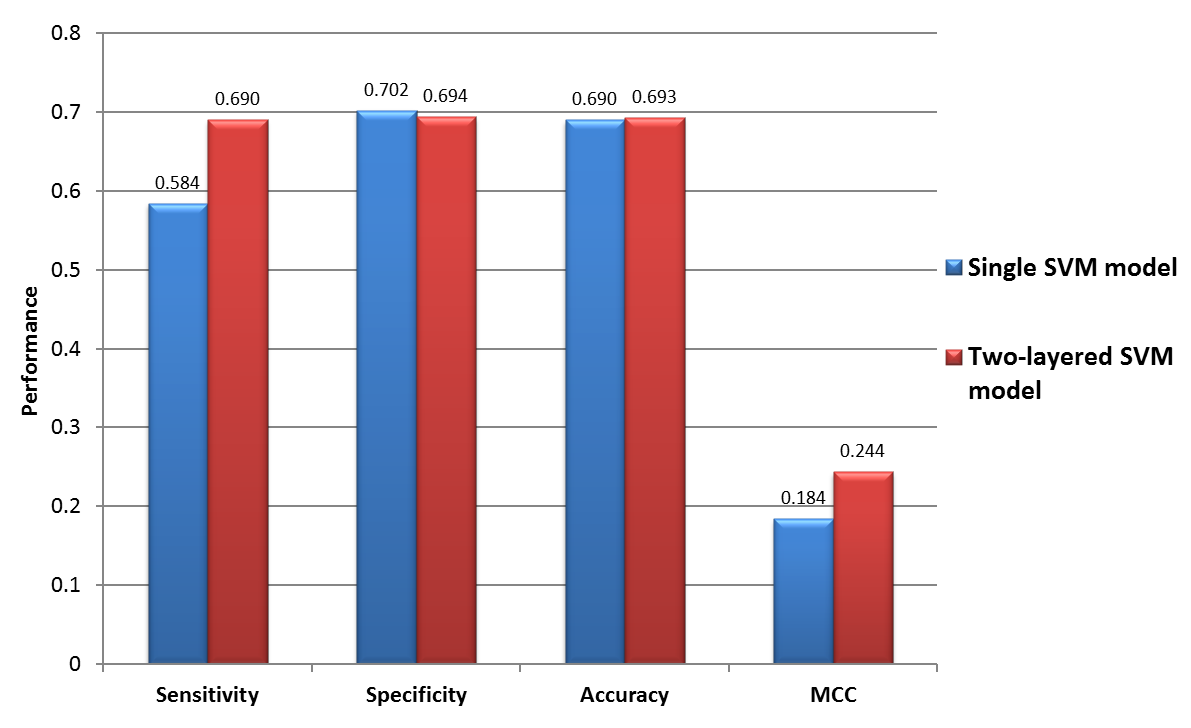

Supplement: S7 Fig — (TIF) [file pone.0179529.s007.tif]

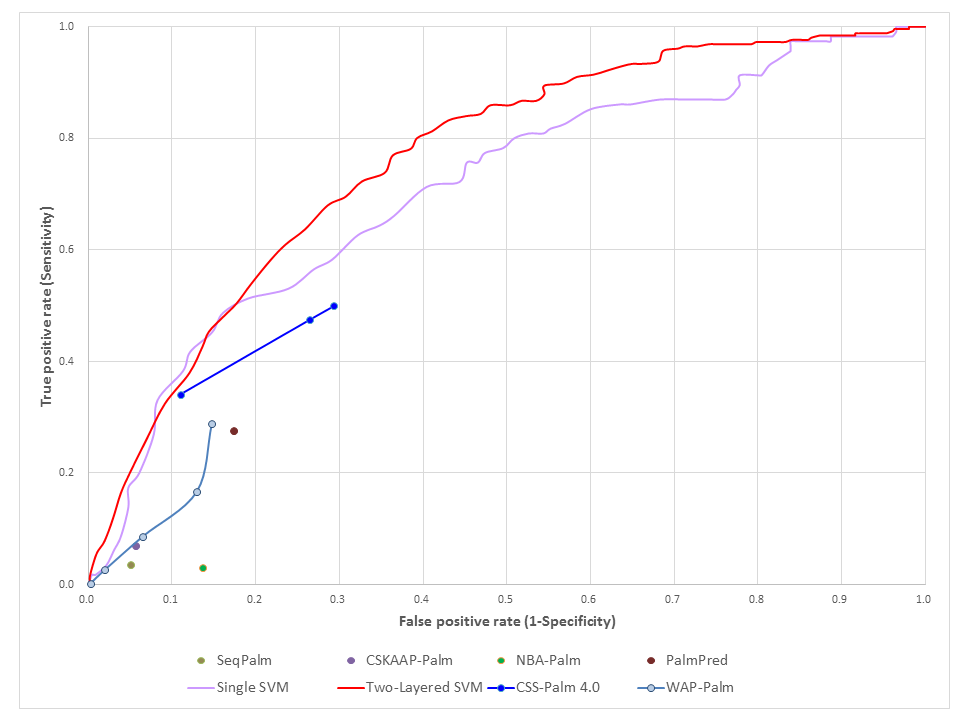

Supplement: S8 Fig — (TIF) [file pone.0179529.s008.tif]
