# Supplementary material for: MDD-Palm: Identification of protein S-palmitoylation sites with substrate motifs based on maximal dependence decomposition
Source: PLoS One. 2017 Jun 29;12(6):e0179529. doi: 10.1371/journal.pone.0179529 (PMC5491019; doi:10.1371/journal.pone.0179529)
Supplement: S2 Table — (DOCX) [file pone.0179529.s010.docx]

**Table S2. Four-fold cross validation results on single SVM model trained with various features.** Sn, sensitivity; Sp, specificity; Acc, accuracy; MCC, Matthews Correlation Coefficient; AUC, area under the curve of ROC.

| **Training features** | **Sn** | **Sp** | **Acc** | **MCC** | **AUC** |
| --- | --- | --- | --- | --- | --- |
| 20D Binary code(AA) | 0.60 | 0.60 | 0.60 | 0.14 | 0.62 |
| BLOSUM62 | 0.63 | 0.61 | 0.61 | 0.17 | 0.63 |
| Amino Acid Composition(AAC) | 0.63 | 0.68 | 0.67 | 0.23 | 0.67 |
| Amino Acid Pair Composition(AAPC) | 0.50 | 0.61 | 0.60 | 0.08 | 0.56 |
| Accessible Surface Area(ASA) | 0.56 | 0.53 | 0.54 | 0.08 | 0.56 |
| Position Weight Matrix(PWM) | 0.61 | 0.67 | 0.66 | 0.21 | 0.65 |
| Position-specific scoring matrix(PSSM) | 0.62 | 0.59 | 0.59 | 0.14 | 0.62 |
| AAC + AA | 0.63 | 0.69 | 0.67 | 0.29 | 0.71 |
| AAC + B62 | 0.62 | 0.60 | 0.61 | 0.16 | 0.63 |
| AAC + AAPC | 0.66 | 0.64 | 0.64 | 0.21 | 0.65 |
| AAC + ASA | 0.63 | 0.60 | 0.60 | 0.16 | 0.63 |
| AAC + PWM | 0.62 | 0.69 | 0.68 | 0.28 | 0.72 |
| AAC + PSSM | 0.70 | 0.69 | 0.69 | 0.32 | 0.75 |
