# Supplementary material for: MDD-Palm: Identification of protein S-palmitoylation sites with substrate motifs based on maximal dependence decomposition
Source: PLoS One. 2017 Jun 29;12(6):e0179529. doi: 10.1371/journal.pone.0179529 (PMC5491019; doi:10.1371/journal.pone.0179529)
Supplement: S5 Table — (DOCX) [file pone.0179529.s013.docx]

**Table S5. Ten-fold cross validation results on single SVM model trained with various features.** Sn, sensitivity; Sp, specificity; Acc, accuracy; MCC, Matthews Correlation Coefficient; AUC, area under the curve of ROC.

| **Training features** | **Sn** | **Sp** | **Acc** | **MCC** | **AUC** |
| --- | --- | --- | --- | --- | --- |
| 20D Binary code (AA) | 0.67 | 0.68 | 0.68 | 0.26 | 0.68 |
| BLOSUM62 (B62) | 0.67 | 0.68 | 0.68 | 0.26 | 0.68 |
| Amino Acid Composition (AAC) | 0.62 | 0.63 | 0.63 | 0.18 | 0.60 |
| Amino Acid Pair Composition (AAPC) | 0.63 | 0.66 | 0.65 | 0.22 | 0.64 |
| Accessible Surface Area (ASA) | 0.61 | 0.65 | 0.64 | 0.20 | 0.62 |
| Position Weight Matrix (PWM) | 0.68 | 0.69 | 0.69 | 0.29 | 0.70 |
| Position-specific scoring matrix (PSSM) | 0.67 | 0.68 | 0.68 | 0.26 | 0.68 |
| AAC + AA | 0.70 | 0.69 | 0.69 | 0.31 | 0.73 |
| AAC + B62 | 0.71 | 0.70 | 0.70 | 0.33 | 0.75 |
| AAC + AAPC | 0.74 | 0.74 | 0.74 | 0.40 | 0.80 |
| AAC + ASA | 0.68 | 0.69 | 0.69 | 0.29 | 0.70 |
| AAC + PWM | 0.72 | 0.73 | 0.73 | 0.37 | 0.78 |
| AAC + PSSM | 0.74 | 0.74 | 0.74 | 0.40 | 0.80 |
